# A cross-sectional study examining the nature and extent of interprofessional education in schools of pharmacy in the United Kingdom

**DOI:** 10.1007/s11096-023-01655-0

**Published:** 2023-11-03

**Authors:** Clare Depasquale, Scott Cunningham, Sabrina Anne Jacob, Anne Boyter, Jane Portlock, Ailsa Power, Brian Addison

**Affiliations:** 1 School of Pharmacy and Life Sciences, Robert Gordon University, Aberdeen, UK; 2 Strathclyde Institute of Pharmacy and Biomedical Sciences, University of Strathclyde, Glasgow, UK; 3 School of Life Sciences, University of Sussex, Brighton, UK; 4 NHS Education for Scotland, Glasgow, UK

**Keywords:** Education, pharmacy, Interprofessional education, Learning, experiential, Learning, interprofessional

## Abstract

**Background:**

Interprofessional education can prepare the workforce for collaborative practice in complex health and social care systems.

**Aim:**

To examine the nature and extent of interprofessional education in schools of pharmacy in the United Kingdom.

**Method:**

An online questionnaire was developed using systems theory, published literature and input from an interprofessional expert panel; it included closed and open-ended questions and a demographic section. Following piloting, it was distributed to 31 schools of pharmacy. Descriptive statistics were used for quantitative data, and a content analysis approach for qualitative data.

**Results:**

Ten schools of pharmacy responded. All reported delivering compulsory interprofessional education. Most (80%) reported an interprofessional steering group overseeing development. Formative and/or summative assessment varied depending on year of study. Mechanism and purpose of evaluation varied with respondents reporting Kirkpatrick Evaluation Model Levels 1–3 (100%; 80%; 70%). Two themes were identified: “Variation in Interprofessional Education Approaches and Opportunities” and “Factors Influencing Development and Implementation of Interprofessional Education”. Formal teaching was mainly integrated into other modules; various pedagogic approaches and topics were used for campus-based activities. Respondents referred to planned interprofessional education during practice-based placements; some still at pilot stage. Overall, respondents agreed that practice-based placements offered opportunistic interprofessional education, but a more focused approach is needed to maximise student pharmacists’ learning potential.

**Conclusion:**

Most interprofessional education offered in undergraduate pharmacy curricula in the United Kingdom is campus-based; the nature and extent of which varies between programmes. Very few examples of practice-based activities were reported. Results may inform future interprofessional education curricular development.

**Supplementary Information:**

The online version contains supplementary material available at 10.1007/s11096-023-01655-0.

## Impact statements

- Valuable but variable campus-based interprofessional education is included in undergraduate pharmacy programmes in the United Kingdom indicating the need for further development.
- Highlights the need for increased focus on preparation and planning to maximise student pharmacists’ learning from opportunistic interprofessional education during practice-based placements.

## Introduction

Increased complexity in delivery of care associated with an ageing population and prevalence of long-term chronic diseases has challenged health and social care systems worldwide, acting as an impetus for change in professional practice. It has led to a transition from compartmentalised care involving hierarchical provider-patient dyads to person-centred care focusing on holistic care provision, underpinned by an ethos of collaboration [1, 2]. This paradigm shift has resulted in increasing global recognition of the important role collaborative models of practice play in the delivery of safe, effective, and efficient person-centred care. In turn, this has increased focus on collaborative models of education, strengthening the view that interprofessional education (IPE) acts as the foundation to collaborative practice [3]. This is based on the assumption that learning together will better prepare health and social care professionals to work together [4, 5]; of note published literature also highlights the potential negative influence that the hidden curriculum may have on students’ perceptions of collaborative practice [6, 7]. Defined as “occasions when two or more professions learn with, from and about each other to improve collaboration and the quality of care”, IPE is considered essential in shaping a future “collaborative practice-ready” workforce [4, 8]. It is viewed as cultivating “mutual awareness, trust and respect, countering ignorance, prejudice and rivalry in readiness for collaborative practice” to develop learners who demonstrate transferable core competencies essential for collaborative practice [9, 10].

### The pharmacy context

IPE is particularly relevant when viewed in the context of the evolving role of the pharmacist, with delivery of care increasingly shifting from product-centred to person-centred care [11–14]. With even more roles expanding into areas of clinical practice, there is increasing focus on the valuable input as experts in medicines, that pharmacists make to the interprofessional team and patient care [15–18]. Internationally, regulators overseeing initial education and training of healthcare professionals endorse the importance of interprofessional collaborative practice in health and social care systems and have increased requirements in undergraduate curricula to ensure students have IPE opportunities [19, 20]. In the pharmacy context, examples include the United States (US) standards and the United Kingdom (UK) General Pharmaceutical Council (GPhC) standards specifying the inclusion of IPE opportunities; commencing at an early stage in the Master of Pharmacy (MPharm) programme and progressively developing throughout the years of study to provide opportunities that “mirror practice” [14, 21].

### IPE development in pharmacy education

The “Interprofessional Education in a Pharmacy Context: Global Report” presents a collection of case studies highlighting diverse and innovative IPE approaches used internationally in the context of pharmacy education [16].

Two studies exploring IPE offered in colleges and schools of pharmacy in the US report heterogenous campus and practice-based initiatives involving different professional groups, adapting different pedagogical approaches and methods of assessment and evaluation [22, 23]. Patel et al. used telephone interviews and reflective accounts to gather a snapshot of IPE in UK schools of pharmacy. The authors report a variable degree of IPE delivery. The type of activities undertaken varied considerably, with the authors noting a range from multiprofessional to truly interprofessional learning opportunities [24]. These studies highlight the lack of standardisation in IPE and several facilitators and barriers to its development and implementation. It is currently unclear whether the requirement for increased IPE articulated in the new GPhC standards has intensified the development and implementation of IPE opportunities in UK MPharm programmes [14].

### Aim

The aim of this study was to explore the nature and extent of IPE currently offered in MPharm programmes across the UK.

### Ethics approval

Ethical approval was granted by the Robert Gordon University School of Pharmacy and Life Sciences Ethics Review Committee (Approval Number S291) in April 2021.

## Method

### Research design

Considering the complex nature of IPE, the research team have drawn upon a pragmatic research paradigm informed by systems theory which follows the principle of thinking about things as a whole rather than in parts [25]. An online survey methodology was employed using a questionnaire including both closed and open-ended questions. This aimed to maximise the potential of the collected data to answer the research aim.

### Data collection

Initial development of the questionnaire was informed by a literature review, the Biggs 3P Model and the 3P Model of Learning to Collaborate [26, 27]. The two models were used to provide an overall framework, with questions aligned to the components included in the 3Ps—presage, process, product. To ensure content validity, further development included five phases of discussion, review and modification by the research team and circulation to an interprofessional four-member expert panel. Members involved in the development of the questionnaire included representation from the Centre for the Advancement of Interprofessional Education (CAIPE), the medical and nursing professions and academic staff from four schools of pharmacy geographically distributed throughout the UK.

The Online Surveys Tool was used to develop the questionnaire [28]. Free text boxes were used for open-ended questions and binary response (yes/no) or multiple-choice response options for closed questions. A demographics section was included. The questionnaire included 19 questions grouped into four sections: the structure of IPE (3 questions), the nature of IPE activities on offer (6 questions), the evaluation of IPE activities (5 questions), demographics (5 questions).

### Sampling

In May 2021, the questionnaire was piloted for usability and participant understanding with three members of academic staff. A whole population sampling strategy was employed for questionnaire distribution. An email was sent to a key member of academic staff at all 31 schools of pharmacy (SoPs) in the UK including the two in Scotland, in June 2021, inviting them to participate in the study. An initial list of staff used in previous research, was updated through verification on university websites. Three reminders were sent over a 12-week period to encourage a better response rate. Multiple responses from each SoP were not sought.

### Data analysis

Quantitative data generated from closed questions were analysed using descriptive statistics. A content analysis approach was used for data generated from open-ended questions by one member of the research team (CD) [29]. This was independently verified by another member (BA). The Biggs 3P Model was used as the framework for data analysis. Additional components identified in the 3P Model of Learning to Collaborate were considered during the coding phase (Table 1) [26, 27].

**Table 1:** Content analysis phases

Familiarisation: Reading and re-reading the individual responses to gain a general understanding of the overall data collected.
Condensation: With the study aim and objectives in mind, selection, and extraction of the relevant text from each SoP response.
Coding: Linking the text to components (used as the coding categories/sub-categories) presented in the 3P Model of Learning to Collaborate [27].
Categorisation; Grouping together components in the 3Ps—Presage, Process and Product.
Theme Development: Condensing data into themes aligning with the research aim and objectives.

## Results

Ten SoPs (32%) responded. Table 2 lists the academic roles of respondents.

**Table 2:** Respondent’s role in school of pharmacy (n = 10)

IPE champion/co-ordinator	5
Director of teaching & learning	2
MPharm IPE lead/deputy lead for IPE steering Group	1
Lead for IPE and PPE	1
Head of school	1

### Quantitative results

Respondents reported various healthcare professional programmes taught at their institution, with 60% (n = 6) offering pharmacy, medicine, and nursing programmes.

A wide range of professional disciplines were included in campus-based activities—medicine, nursing, midwifery, dentistry, physiotherapy, occupational therapy, dietetics, speech and language therapy, psychology, physician associate, dental and hygiene therapy, diagnostic radiography, biomedical science, paramedic science, food and nutritional science, social care, law, NHS technicians. Contrastingly, those disciplines involved in planned practice-based IPE activities were limited to medicine, nursing and podiatry. Disciplines not involved in either campus-based or practice-based IPE included veterinary science and teaching.

Most respondents (n = 8; 80%) reported an interprofessional steering group overseeing the development of IPE initiatives; additionally, 90% (n = 9) reported that IPE content was based on the CAIPE “Interprofessional Education Guidelines” [9]. All ten respondents reported that IPE delivery was a compulsory requirement; one SoP offered additional voluntary activities, another both additional voluntary and elective activities. The requirement and nature of activities that student pharmacists had to complete before participating in IPE initiatives varied with 50% (n = 5) of respondents reporting student pharmacists completed an individual online activity; other approaches included completion of an internally developed pre-activity survey (n = 1; 10%), pre-reading (n = 1; 10%) and communication with group members before the actual IPE session (n = 1;10%).

Most respondents used formative and summative assessment with approaches varying depending on the year of study. Staff feedback was the most used approach for formative assessment, followed by peer feedback. One respondent referred to patient educator feedback and a reflective exercise completed in third year. Summative assessment methods included reflective exercises, group presentation, portfolio, and Objective Structured Clinical Examinations (OSCEs) (Table 3).

**Table 3:** Assessment approaches (n = 10)

Year of study	Formative assessment	Summative assessment
Year 1	Staff feedback (n = 8)	Reflective exercise (n = 4)
	Peer feedback (n = 3)	Group presentation (n = 1) Portfolio (n = 1) OSCE (n = 1)
Year 2	Staff feedback (n = 8)	Reflective exercise (n = 5)
	Peer feedback (n = 4)	Group presentation (n = 1) Portfolio (n = 2) OSCE (n = 1)
Year 3	Staff feedback (n = 7)	Reflective exercise (n = 4)
	Peer feedback (n = 2)	Portfolio (n = 2)
	Patient educator feedback/Reflective exercise (n = 1)	OSCE (n = 2)
Year 4	Staff feedback (n = 8)	Reflective exercise (n = 5)
	Peer feedback (n = 2)	Group presentation (n = 1) Portfolio (n = 2) OSCE (n = 3)

A variety of mechanisms was used to evaluate IPE activities. These included published/validated student surveys (n = 1; 10%), internally developed student surveys (n = 10; 100%), student verbal feedback (n = 5; 50%), academic staff/facilitator surveys (n = 3; 30%), academic staff/facilitator reports (n = 3; 30%), academic staff/facilitator verbal feedback (n = 8; 80%) and student reflective statements (n = 1; 10%). The purpose of evaluation reported by respondents is included in Table 4.

**Table 4:** Purpose of evaluation

Levels 1–4 in the Kirkpatrick evaluation model [44]	Number of responses
	(n);(%)
Level 1 Reaction: To evaluate how well IPE activities and initiatives were received by students.	n = 10; 100%
Level 2 Learning: To evaluate how effective IPE activities and initiatives were in terms of increasing student knowledge and understanding (collaborative competencies).	n = 8; 80%
Level 3 Behaviour: To evaluate how IPE activities and initiatives have altered student beliefs, perceptions and behaviours (collaborative competencies).	n = 7; 70%
Level 4 Results: To evaluate how IPE activities have impacted patient outcomes.	n = 0

### Qualitative results

Two main themes were identified from responses to open-ended questions. These are presented in a narrative description linked to components in the Biggs 3P Model and the 3P Model of Learning to Collaborate [26, 27].

### Theme 1: variation in interprofessional education approaches and opportunities

#### 3P: PROCESS—approaches to learning and teaching: formal/informal learning; campus-based/practice-based learning

Formal teaching/learning was mainly delivered integrated in other modules. Various pedagogic approaches were used for activities delivered on campus to varying extents across all 4 years of the curriculum. These included simulation, online group learning, blended group learning—classroom/online based group discussion and classroom/online case-based discussion, problem-based learning, student–student peer teaching and IPE conferences. Topics included in campus-based IPE activities were patient safety, medication safety, Human Factors/systems thinking, mental health, person-centred care, ethical dilemmas, public health, cultural awareness, specialist clinical areas, values-based practice, communication skills and collaborative practice. Other topics included numeracy skills and professional negligence.

Some respondents referred to planned practice-based IPE activities; these involved second and third year medical and nursing students, second year podiatry students and third year medical students with some initiatives still at pilot stage. Please refer to supplementary material.

Informal teaching/learning was mainly referred to in the context of placements.

*“Our formal IPE sessions are based at the university. However, in the placements, we encourage our students to find out the role of various practitioners in caring for patients. They also observe how a pharmacist interacts within a multidisciplinary team”*. (SoP1)

Overall, respondents agreed that practice-based experiential learning placements provided many opportunities for unplanned IPE. However, more preparation and planning may be required to ensure opportunities are not missed; one response referred to the importance of student pharmacists identifying and acting on these opportunities.

*“Students on our placements in the future will be required to actively seek these opportunities/collaborative moments. Evidence for their portfolio would be required”.* (SoP4)

Another respondent raised the issue of the equitable nature of these unplanned IPE opportunities.

*“Lots, this is encouraged, but the problem is that this is an uneven experience, so* [some] *placements have lots and others don't, depending on the nature of the placements”.* (SoP9)

Again, there was a varied response to how much time was allocated to campus-based IPE in the MPharm programme. This ranged from 1.5 to 9+ hours in first year to 4 to 15 h in the final year of study (Table 5).

**Table 5:** Timetabled campus-based IPE

School of Pharmacy (SoP)	Year of study			
	Year 1 (Hours)	Year 2 (Hours)	Year 3 (Hours)	Year 4 (Hours)
SoP 1	9 + hours they spend working together to create a presentation	5 + pre-work which is 10 h	6	4
SoP 2	4	9	9	15
SoP 3	9	9	9	4
SoP 4	4	2	8	8
SoP 5	3 (1 cross programme event; 1 bespoke event with nursing)	There have been 90 min of communication session/student previously with MBChB but this did not happen last year. Pilot last year carried out to allow 2 h/student cross programme	2	4
SoP 6	4	6	10	14
SoP 7	6	6	4	6
SoP 8	4	3	6	15
SoP 9	4	8	8	6
SoP 10	1.5	1.5	Unknown	Unknown

### Theme 2: factors influencing development and implementation of IPE in MPharm programmes

#### 3P: PRESAGE—context: political climate

Issues were highlighted regarding organisational culture around IPE; the view being that a broader approach is needed both at university level and across education, health, and social care sectors to ensure the success of IPE initiatives.

*“To do IPE properly there needs to be a cross university initiative, allowing a drive to implement across a range of programmes. Otherwise, it is just a piecemeal and sometimes disjointed activity, using what is available rather than what is desirable. Schools and courses can decide to drop in and out when they wish, regardless of the effect on others thus keeping going can be a battle, let alone developing further”*. (SoP9)

#### 3P: PRESAGE—context: regulatory frameworks

Some respondents referred to the requirement by the GPhC articulating the inclusion of IPE initiatives in the undergraduate curriculum.

*“We feel we deliver a rich and varied programme of activities which fully engage our students. My main concern is that the GPhC continue to quantify IPE in simplistic terms of the number of hours students spend together. We firmly believe that it's not amount of time but quality of time that is key - quality, not quantity”.* (SoP7)

#### 3P: PRESAGE—context: funding

Respondents reported challenges encountered during the development and delivery of campus-based initiatives included a lack of resources and funding. Cost was also reported to be a challenge encountered during the development and delivery of practice-based IPE initiatives. A respondent commented that there are opportunities for unplanned IPE in practice-based placements, but those possibilities were dependent on available funding.

*“Yes, there are opportunities* [for unplanned IPE within practice-based placements] *but that is based on the assumption that practice-based placements are a possibility for a SOP where funding and resource is limited”.* (SoP3)

#### 3P: PRESAGE—context: space and time constraints; competing curricular demands

Several respondents referred to challenges in this context both for campus-based and practice-based activities; these included availability of IPE facilitators including practice-based facilitators, increased staff workload and room availability. One respondent commented that several issues were eased through online delivery during the COVID-19 pandemic, however this led to new challenges.

*“Some of these issues were eased through remote delivery in COVID. However online interaction brings different challenges”.* (SoP5)

Challenges around logistics and timetabling were mentioned by most respondents for developing/delivering campus-based and practice-based activities.

#### 3P: PRESAGE—teacher/programme developer characteristics: conceptions of learning and teaching; conceptions of collaboration; learner perceptions; enthusiasm

A challenge identified by respondents during the development and delivery of practice-based initiatives was staff “buy-in” and practice facilitators (pharmacists) resistance to engage with facilitating sessions. A respondent reported that this extended to other disciplines in the clinical environment, due to the perceived impact on the experience of other healthcare professional students.

*“Enthusiastic keen practitioners are on board however difficult to persuade those tutors with less experience to take this on”*. (SoP5)

*“Medical programme not keen to support IPE in the practice environment as it is perceived that this would reduce the learning value of their students”*. (SoP3)

#### 3P: PRESAGE—teacher/programme developer characteristics: teacher’s expertise

The nature and extent of training offered to academic staff, practice-based facilitators and student facilitators varied. Several respondents replied “not applicable” to the latter as peer-teaching was not included in their IPE programme. The different approaches to training provision for academic staff ranged from synchronous online training, briefings, face-to-face training, tailored training to individual IPE sessions, facilitator guides, training video to no training at all. Training provided to practice-based facilitators was more limited.

*“None, other than shadowing other staff if they are new members of staff”.* (SoP8)

## Discussion

### Key findings

This study explored the nature and extent of IPE in MPharm programmes with the aim of providing an up-to-date overview of current activities offered to student pharmacists by SoPs in the UK. Study results present a varied picture, setting out diverse campus-based activities, involving different professional groups with medicine and nursing the most represented. Several pedagogical approaches, methods of assessment and evaluation were adopted. A variety of topics were covered.

All participating SoPs offered compulsory IPE, with time allocated to campus-based activities varying between programmes. Additionally, a picture of limited planned practice-based initiatives currently on offer during placements was presented with results highlighting that placements may present unplanned IPE opportunities. Respondents identified a need for a more focused approach to preparation and planning to ensure that unplanned opportunities are not missed, maximising potential student pharmacist learning. The results corroborate findings from previous studies that there is no standardised approach to IPE delivery and highlights the variable interpretation of what constitutes an appropriate “interprofessional learning plan” as stipulated by the GPhC [14, 22–24].

### Strengths and limitations

A strength is the methodological approach underpinned by theory. Inclusion of open-ended questions allowed a deeper insight into the nature of IPE initiatives and better understanding of barriers influencing curricular campus-based and practice-based IPE development. There are limitations. The study only included SoPs in the UK; therefore, the research findings may lack generalisability and transferability to other countries. A limitation inherent to survey design—non-response bias must be considered. Despite several factors being taken into consideration—minimising respondent burden, appropriate timing for distribution to avoid the busy academic calendar, extended period of data collection and several reminder emails, the response rate was low. Therefore, the data provided may not be reflective of the wider UK context as responses from SoPs that did not participate could differ from responses collected. A similar study undertaken by Patel et al. exploring IPE in UK SoPs elicited a similar response rate [24]. It triggers thought as to reasons for lack of engagement with the subject area. Bearing in mind the complex nature of IPE, with responsibilities often shared between academic staff, this factor could have impacted on ownership of the need to respond. However, despite this study not providing a complete overview of IPE delivered in all SoPs in the UK, it does add to the limited data emerging from the UK by providing a more current update on IPE offered in MPharm programmes.

### Interpretation

The literature refers to the complementary nature of campus-based activities and practice-based activities, with the former considered an opportunity to prepare students for IPE in practice. By introducing foundational IPE concepts and opportunities, campus-based activities allow students to engage collaboratively in immersive experiences that mirror professional practice in a safe and supportive learning environment [7, 9]. Published literature also highlights the need for contextualisation to local needs when developing IPE initiatives [8, 16]. Unlike medical and nursing pre-registration programmes, where practice-based placements almost eclipse campus-based teaching/learning throughout the programme, student pharmacists in the UK are presented with limited practice-based opportunities over the initial 4 years of study. The 4-year MPharm programme is followed by a foundation year in practice and completion of the registration assessment prior to eligibility to become a registered pharmacist. While the results of this study show that campus-based IPE is available to varying extents in MPharm programmes, it also highlights that increased focus on IPE through campus-based activities could potentially be viewed as an effective model to adequately prepare student pharmacists to actively seek out IPE opportunities while on placement, enabling them to take advantage of unplanned interprofessional learning opportunities [30, 31]. It may be argued that these unplanned IPE experiences do not conform to the criteria set out in CAIPE’s definition of IPE, however, others hold the view that “no matter how limited learning opportunities for collaborative practice are, they do exist, and we have to ensure that students have the opportunity to work alongside and with other professions” [32, 33].

Furthermore, the literature refers to the interprofessional “learning continuum” that encompasses learning commencing at undergraduate level and continuing through postgraduate opportunities in the work-place setting [3, 4, 34–38]. Additionally, the Interprofessional Education Guidelines stress the importance of collaboration between higher education institutions and practice placement providers to strengthen interprofessional opportunities [9]. Viewing this in the UK context, with an increasingly integrated approach between the university curriculum and the foundation year articulated in the GPhC standards, the foundation year where trainee pharmacists are placed in the workplace, could present a practical approach for organised IPE initiatives [14]. This could help overcome limitations encountered by some higher education institutions that do not offer certain health and social care programmes [39].

The development and implementation of IPE initiatives in undergraduate curricula is not without its challenges [40]. Qualitative data collected from respondents in this study supports previous findings and reports several barriers to IPE curricular development. One respondent referred to the transition to virtual delivery during the COVID-19 pandemic as a way of overcoming some of these barriers; what Langlois et al. refer to as the pandemic “silver lining”, catapulting higher education institutions into adapting IPE opportunities to virtual delivery [39]. This move away from traditional face-to-face models is viewed as one way in overcoming logistical challenges due to timetabling issues, space constraints due to large student cohorts from multiple programmes and lack of geographically co-located programmes. However, as identified in this study, virtual and hybrid modes of delivery may themselves present new challenges; several authors articulate a need for proper planning to review the most appropriate pedagogical approaches and topics suited to online teaching/learning as well as adequate staff/facilitator training to ensure effective student learning [39, 41–43].

## Conclusion

This study highlights that most IPE offered in UK SoPs is campus-based; the nature and extent of which varies between MPharm programmes. Respondents referred to planned IPE during placements, some still at pilot stage. Overall, respondents agreed that placements offered opportunistic IPE, but more focus is needed to maximise student pharmacists’ learning potential. Results from this study could be used to inform future IPE development and implementation.

### Supplementary Information

Below is the link to the electronic supplementary material.

Supplementary file1 (DOCX 14 KB)
