# Supplementary material for: A cross-sectional study examining the nature and extent of interprofessional education in schools of pharmacy in the United Kingdom
Source: Int J Clin Pharm. 2023 Nov 3;46(1):122–30. doi: 10.1007/s11096-023-01655-0 (PMC10830770; doi:10.1007/s11096-023-01655-0)
Supplement: Supplementary file 1 — Supplementary file1 (DOCX 14 KB) [file 11096_2023_1655_MOESM1_ESM.docx]

Supplementary Materials

Examples of Planned Campus-Based IPE Activities

*“Numeracy skills in Year 1 with medical students/nursing students”.*

*“Year 1 Human Factors workshop with nursing – case-based discussion”.*

*“PK* [pharmacokinetics] *case studies in Year 2 with Year 1 medical students”.*

*“Primary care simulations with Year 3 pharmacy and medical students”.*

*“Professional negligence cases in a mock court room with trainee barristers and Year 3 pharmacy students”.*

*“In Year 2, management of a particular therapeutic area, in this case pain management, is explored in an interprofessional setting using virtual patients”.*

*“Year 3 case discussion around medicines administration MPharm and Nursing”.*

*“Classroom/online based group discussion, classroom/online case-based discussion; Year 1 Professional Values; Year 2 Safety and Risk – A Team Approach”.*

*“Case-based learning with medical students in a team of 6-8 on a scenario”.*

*“Year 4 pharmacy and physiotherapy students where physiotherapy students teach pharmacy students chest auscultation and pharmacy students teach physiotherapy students inhaler technique and peak flow plus respiratory therapeutics”.*

*“Year 4 pharmacy and dentistry students with case-based discussion around dental health”.*

*“Interprofessional ethics problem-based learning with final year pharmacy and nursing students”.*

*“Medicines governance problem-based learning with final year pharmacy, medical and nursing students”.*

*“Our final year students work with physiotherapy and diagnostic radiography students on clinical cases”.*

*“In Year 4 medication errors are discussed using ‘real’ medication incidents which have occurred in healthcare and students undertake a root cause analysis to develop preventative strategies through collaborative working”.*

*“IPE conferences in Years 1 and 4 which included workshops and interprofessional interactions”.*

**Examples of Planned Practice-Based IPE Activities**

*“Students visiting a podiatry hospital and undertaking work on patient cases with podiatry students”.*

*“Year 3 MPharm with Year 1 MBChB in Primary Care (1 day) where MPharm students work with the GP tutor to facilitate discussion around medicine taking (not available currently for all students)”.*

*“We have done one small pilot where a small group of our students worked with medical and nursing students who were also on placement and reviewed patients together focusing on adherence and polypharmacy”*.
